# 3D Multi-Microchannel Helical Mixer Fabricated by Femtosecond Laser inside Fused Silica

**DOI:** 10.3390/mi9010029

**Published:** 2018-01-16

**Authors:** Chao Shan, Feng Chen, Qing Yang, Zhuangde Jiang, Xun Hou

**Affiliations:** 1 State Key Laboratory for Manufacturing System Engineering and Key Laboratory of Photonics Technology for Information of Shanxi Province, School of Electronics & Information Engineering, Xi’an Jiaotong University, Xi’an 710049, China; shanchaobest@stu.xjtu.edu.cn (C.S.); houxun@mail.xjtu.edu.cn (X.H.); 2 The International Joint Research Center for Micro/Nano Manufacturing and Measurement Technologies, Xi’an Jiaotong University, Xi’an 710049, China; yangqing@mail.xjtu.edu.cn (Q.Y.); zdjiang@mail.xjtu.edu.cn (Z.J.); 3 School of Mechanical Engineering, Xi’an Jiaotong University, Xi’an 710049, China

**Keywords:** passive micromixer, femtosecond laser, microstructure fabrication

## Abstract

Three-dimensional (3D) multi-microchannel mixers can meet the requirements of different combinations according to actual needs. Rapid and simple creation of 3D multi-microchannel mixers in a “lab-on-a-chip” platform is a significant challenge in micromachining. In order to realize the complex mixing functions of microfluidic chips, we fabricated two kinds of complex structure micromixers for multiple substance mixes simultaneously, separately, and in proper order. The 3D multi-microchannel mixers are fabricated by femtosecond laser wet etch technology inside fused silica. The 3D multi-microchannel helical mixers have desirable uniformity and consistency, which will greatly expand their utility and scope of application.

## 1. Introduction

Micro-total analysis systems and “lab-on-a-chip” platforms are widely used for sample preparation and analysis, drug delivery, and biological and chemical synthesis [1,2,3,4,5]. As essential components of many microfluidic systems, micro-mixing devices are used to homogenize samples or reactions. With the development of microfluidic systems, numerous micromixers based on kind of material have been reported [6,7,8,9,10].

At present, the application of microfluidic chips allows micromixers to mix only two substances at once, hardly meeting new requirements. Therefore, new micromixers that can mix multiple substances simultaneously or separately are needed. Sometimes, mixing substances in the proper order is also required. In chemistry and biology, there is a lot of demand for these more complex micro-mixers. Especially in the area of biological cell culture, cells are often mixed with a variety of nutrient solutions [11]. There are also greater requirements for the micromixer function. Complex structure design can realize complex functions. Therefore, it is necessary to fabricate a multi-microchannel mixer to meet the requirements of different combinations.

Microfluidic devices are not only prepared in fused silica, but also are processed on polymer materials [12,13]. Polymer materials have poor stability and resistance to acid and alkali. Fused silica is an ideal material for microfluidic applications; it has very high optical transparency, low background fluorescence, chemical inertness, and hydrophilicity [14]. Therefore, preparing microfluidic devices inside fused silica can be very effective. However, it is still challenging to fabricate three-dimensional (3D) microfluidic chips in hard/brittle materials like fused silica. Research on micromixer fabrication is crucial to microfluidic chip development.

Micromixers are commonly fabricated by a conventional photolithography and chemical etching process. Planar lithography techniques can be used to prepare some simple micromixers. The reason why this is insufficient for fabricating multichannel micromixers is that the steps of multilayer stacked structure methods become more complicated when the processing structures are asymmetric or complex. Additionally, this approach lacks flexibility. The planar or semi-three-dimensional structure of micromixers limits the mix performance of the devices [15,16]. As a consequence, rapid and efficient mixing is still a challenging task in the design and development of micromixers.

Based on the demand, femtosecond laser micromachining has become a feasible tool to fabricate 3D structures inside transparent hard/brittle materials [17,18]. In a previous study [19], we proposed fabrication of 3D microchannels with arbitrary lengths and uniform diameters in fused silica by femtosecond laser wet etch (FLWE) technology. This simple and flexible technology provides new ideas and solutions for processing 3D multi-microchannel mixers in fused silica.

Compared with previous work, the difficulty of multichannel micromixer processing mainly exists in device uniformity, integration level, and stability. The channel diameters of single micromixers fabricated in previous work have inhomogeneity of several microns. The defect of the encapsulation process also makes the mix process fail often. In order to address these deficiencies, we took a variety of approaches: adopted more accurate dynamic laser power regulation and two-step wet etch method for device uniformity; adopted the method of oxygen plasma assisted encapsulation for device stability; and designed two different structures for different mix requirements. We also paid more attention to practical applications of 3D helical mixers and the device properties and functionality.

With these approaches, we fabricated 3D multi-microchannel helical mixers with good uniformity and consistency inside fused silica based on FLWE technology. The design and improved micromixer structure can extend the performance of the chip to meet diverse needs. This work addresses the two main problems with creating feasible 3D multi-microchannel mixers: (1) effectively improving the device’s integrality; and (2) developing a robust but simple packaging method to connect the 3D micromixers to the external devices. In addition, the utility and scope of application of the micromixers fabricated by FLWE are greatly improved.

## 2. Materials and Methods

**Figure 1:**
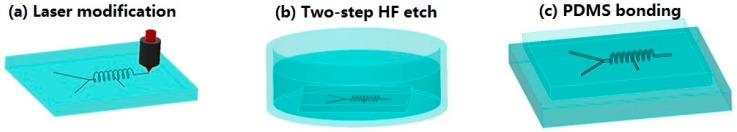
Schematic diagram of the fabrication process. (**a**) Laser modification with accurate dynamic laser power regulation; (**b**) 5% HF wet etch for 1 h and 10% HF wet etch for 2 h; and (**c**) oxygen plasma assisted encapsulation.

The micromixer manufacturing process includes two steps: the laser writing process and the chemical wet etch, as shown in Figure 1. The femtosecond laser micromachining system used to fabricate the micromixer involves a femtosecond laser source (wavelength: 800 nm, pulse duration: 50 fs, repetition rate: 1 kHz), a microscope objective (NA = 0.9, 100×, Nikon, Tokyo, Japan), a programmable three-axis stage (H101A ProScan II Upright Stage, Prior Scientific, Cambridge, United Kingdom), a charge-coupled device camera (Nikon), and a laser beam control system. The helical micromixer was fabricated embedded in the fused-silica substrate (1.0 cm × 1.0 cm × 0.9 mm). The channel was written by the laser by moving the 3D stage along the pattern path at a speed of 10 μm/s. The laser power was adjusted from 3 mW to 7 mW by a computer-controlled attenuator with temporal modulation of the power compensation. Instead of linearly polarized laser light, circularly polarized laser light is used in the manufacture of complex 3D microchannels with a high etching rate [20]. The geometry of the channel, the height, and the pitch circle diameter of the helix can be controlled by computer.

Second, we used two-step wet etch. The mixer sample was immersed in an ultrasound-assisted solution of 5% hydrofluoric acid (HF) for 1 h. We call this process pre-etching. This allows the entire femtosecond laser–irradiated pattern to have sufficient, but not intense, contact with HF. Then we put this sample into another solution of 10% HF for about 2 h, and a hollow spiral channel with a dimension of 40 μm was obtained. With this approach, it is possible to improve the uniformity and smoothness of the microchannel’s inner wall to reduce the flow resistance of liquid materials.

To create the connector for the injection process, a fused-silica sample was placed on a prepared polydimethylsiloxane (PDMS) film. We optimally treated the PDMS substrate and the silica chip with oxygen plasma for 80 s, and then bonded them together under room temperature conditions. Finally, two syringe needles were inserted into the PDMS film at the entrance of the channel to connect all the channels. Thus, all the channels will penetrate as integrated.

## 3. Results

In order to realize the complex mixing function for multiple substance mixes simultaneously, separately, or in proper order, we designed and prepared two complex structure micromixers.

### Parallel Mixer

**Figure 2:**
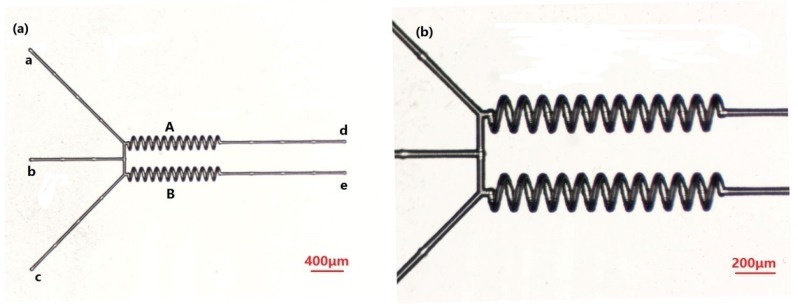
(**a**) Morphology of a multiple channel mixer, observed via optical microscope; and (**b**) detail of mixer with enlarged scale.

The first structure, shown in Figure 2, is composed of multiple channels to mix substances simultaneously or separately. The structure of the micromixer group is made up of two helical microchannel structures (A, B), three inlets (a, b, c), two outlets (d, e), and many microchannels. For the helical microchannel, the number of turns, length, helical pitch, circle diameter, and depth of the helix axis with respect to the sample surface are 12, 1200, 100, 150, and 175 μm, respectively. The diameter of the cross-section is 35 ± 1 μm. In previous studies, there was inhomogeneity of several microns in size. However, in this work, the size error of a single helical structure can be controlled to be between 1 and 2 μm, which greatly improves the uniformity of the device. Meanwhile, the size error of multiple helical structures can be controlled to be between 1 and 2 μm, which ensures consistency of the device. This improvement is achieved by adopting more efficient power and accurately adjusting the scanning speed. The power and scanning speed of the machining process are adjusted according to the structure.

The micromixer can complete the mixing of substances (material 1 and material 2) through the two inlets (a and b) in helical microchannel (A). It can complete the mixing of substances (material 2 and material 3) through the two inlets (b and c) in helical microchannel (B). It can also complete the mixing of substances (material 1 and material 3) in helical channel A or B. More importantly, the micromixer can mix three substances simultaneously.

Traditional mixers are usually combined in series. The mixing of various samples is generally implemented through multiple mixers, step by step. This increases the number of microfluidic chip devices and decreases integration. However, with the multichannel micromixer group we prepared, the utilization efficiency of the micromixer can be greatly improved, and the flexibility of the application is also greatly enhanced. This micromixer can complete different mix tasks at the same time, reducing the number of micromixers needed and further simplifying the structure of microfluidic chips.

For the multiple mixers group, as well, integration is an important performance index. Femtosecond laser micro-nano processing technology can improve integration of the microfluidic chip. On the one hand, in previous studies we were able to obtain significantly higher mixing efficiency of the 3D helical mixer than of the straight channel, and also reduce the size of the chip to save space [19]. On the other hand, reducing the spacing between each unit is an important way to improve integration. As can be seen in Figure 1, the spacing between the two spiral channels is only 100 μm.

**Figure 3:**
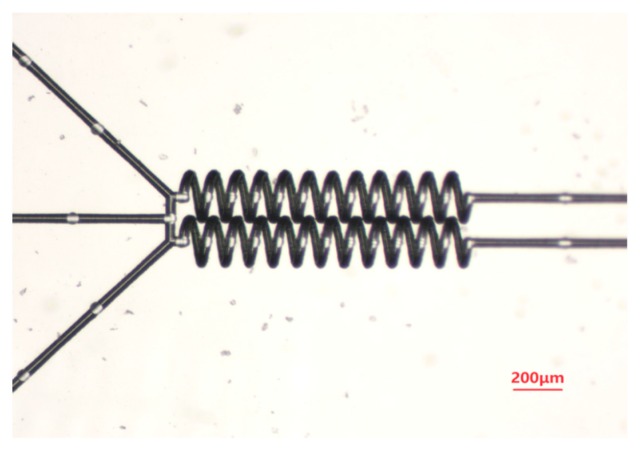
Small spacing structure of a multiple channel mixer.

In order to further show the ability of femtosecond laser micro-nano processing technology to improve the degree of integration, we designed another parallel micromixer like the above structure. We can control the distance between two spiral microchannels to be only a few microns. Greatly reducing the chip size improves integration of the chip. As shown in Figure 3, the space is <5 μm. More importantly, we can integrate multiple micromixers with other devices, such as a micro cabin, or even a metal microstructure [21], to realize more complex functions.

**Figure 4:**
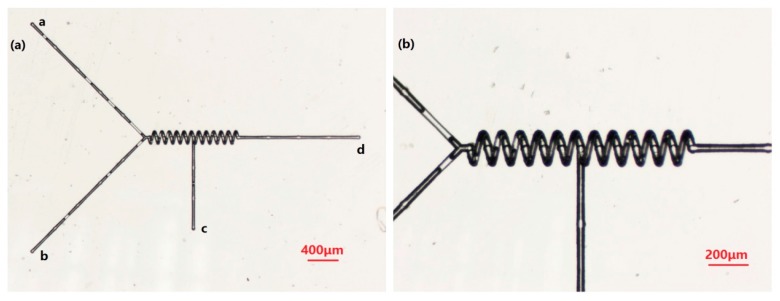
(**a**) Morphology of a multiple channel mixer, observed via an optical microscope; and (**b**) detail of a mixer with enlarged scale.

The second micromixer structure we designed is shown in Figure 4. It is a micromixer used to mix different substances in a particular order. It consists of three inlets (a, b, c), a spiral microchannel, and one outlet (d). For the helical microchannel, the number of turns, length, helical pitch, circle diameter, and depth of the helix axis with respect to the sample surface are 12, 1200, 100, 150, and 175 μm, respectively. The diameter of the cross-section is 35 ± 1 μm.

This mixer can first mix material 1 and material 2 in a spiral microchannel through two inlets, a and b. After mixing evenly, it can continue to mix or react with material 3 in the second half of the spiral microchannel. This step-by-step process has special applications in certain chemical and biological fields. This is very important in the expansion of mix functions. At the same time, we can add new entrances to other parts of the spiral microchannel to meet the multistep mixing function.

## 4. Discussion

**Figure 5:**
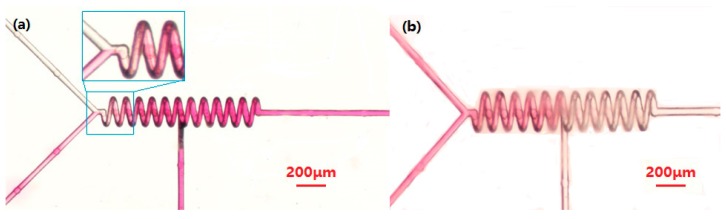
(**a**) Optical micrograph of mixing results of NaOH solution and phenolphthalein solution; (**b**) Mixing results after injecting diluted hydrochloric acid.

We conducted a simple test of the mixing performance of the functional mixer for specific effects of functional mixing. As shown in Figure 5a, NaOH solution (concentration 0.1 M) and phenolphthalein solution (concentration 0.05 M) were injected simultaneously into the two entrances, a and b, respectively, by a syringe pump at a speed of 0.1 mL/h (phenolphthalein becomes pink once mixed with a basic NaOH solution), corresponding to a flow rate of 8.3 cm/s and a low Reynolds number, Re ≈ 2.5. The c inlet did not have any substance injected. As we can see, in the spiral microchannel, the color changes to red, which proves that the two solutions are mixed. As shown in Figure 5b, after the previous step, we injected diluted hydrochloric acid (concentration 0.1 M) into the c inlet. It can be seen in the figure that in the first half of the spiral channel it remained red, while in the second half it became colorless. This proves that sodium hydroxide solution and diluted hydrochloric acid are neutralized and the phenolphthalein becomes colorless, thus proving the mixing effect of the liquid in the second half of the spiral channel.

Finally, the color intensity was obtained from the captured images, pixel by pixel. The intensity of each pixel was normalized. The normalized values correspond to the degree of mixing for each pixel, 0 for unmixed and 1 for fully mixed. The mixing index σ was calculated as:

(1) $$I_{ni}=1-\frac{I_{i}-I_{mix}}{I_{unmix}-I_{mix}}$$

(2) $$\sigma =1-\sqrt{\frac{1}{N}\overset{N}{\underset{n=1}{\sum }}{\left(1-I_{ni}\right)}^{2}}$$

where *I_ni_* is the normalized pixel intensity, *I_i_* is the pixel intensity, *I_unmix_* is the intensity at the unmixed region, *I_mix_* is the intensity at the fully mixed region, and *N* is the number of pixels.

**Figure 6:**
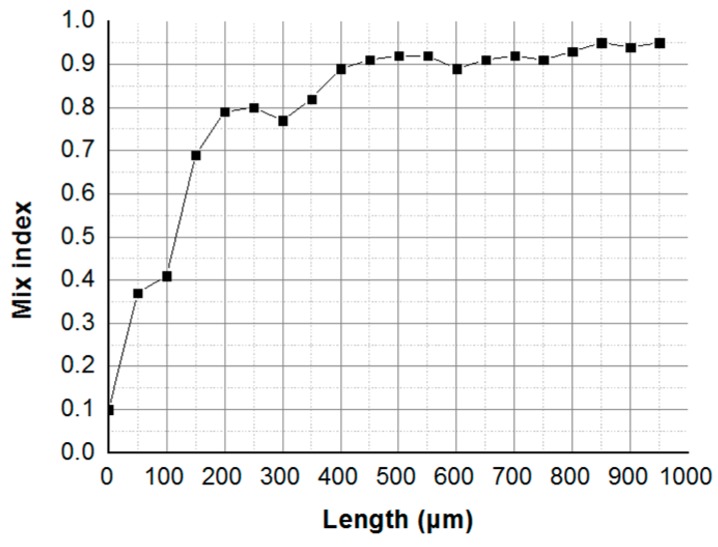
Experimental mixing performance of the helical micromixer: mixing index σ graphics of Figure 5a.

Mixing of the solution is very slow in the upriver straight microchannel. A sharp increase of mixing occurs at the joint of the helical microchannel and the straight microchannel, where a chaotic flow is supposed to arise, as shown in Figure 5a. From the mixing index σ of the normalized pixel intensity *I_ni_*, as illustrated in Figure 6. Here, we define the intersection of the Y-channel as the x-coordinate origin. The straight channel is about 100 μm. Then, full mixing (σ≥0.9) can be verified in 400 μm (at the beginning of the third turn) along the micromixer. The experiment shows that full mixing can be achieved in the helical micromixer quickly and with high efficiency.

## 5. Conclusions

In summary, by means of FLWE technology, some complex 3D multi-microchannel mixers can be achieved inside fused silica. Thus, multi-microchannel mixers with high integration and uniformity for high-performance applications are realized, demonstrating the flexibility and universality of FLWE technology. Meanwhile, a broad spectrum of microfluidics systems fabricated based on these compact and complex 3D multi-microchannel mixers shows promise.
